# Medical News in Paris

**Published:** 1855-01

**Authors:** 


*Medical News in Paris. Letter to the Editors from* R. A. KINLOCH, M. D., of Charleston, S. C.

Mon. Malgaigne, claims some originality in his treatment of fractures; and in his ward, some weeks since, I certainly saw what, to me, was a perfectly novel procedure, in the management of a compound fracture of the leg. The case was one of fracture of both bones, at the most common point, a little below the middle, the fracture of the tibia being oblique. You are aware of the difficulty generally met with in these cases, particularly in managing the upper fragment of the tibia, so as to overcome the anterior angular displacement. The case in point I saw, for the first time, two or three days after the adjustment, which was as follows:—The limb rested upon a double inclined plane, the foot was attached to the foot bound, and there were also lateral splints and pads; no particular counter extension was made use of. But the novel feature in the dressing was the presence of an iron band, which, arching over the member a little above the seat of fracture, passed exterior to the lateral splints, and was secured firmly upon either side of the inclined plane. From the centre of this arch passed a firm steel pin, which could be depressed or elevated at pleasure, its upper two-thirds being wormed, and corresponding with the size of the hole in the band through which it passed. The lower and sharp end of the pin had been made to penetrate the soft tissues, over the flat anterior and inner surface of the upper fragment of the tibia, and was there *firmly fixed in the bony tissue itself.* Thus was this upper fragment kept firmly down in its position, and the angular displacement overcome. There was no evidence of swelling, or other appearances of inflammatory action about the point where the pin penetrated; and the patient assured us that it gave him no particular inconvenience. I watched the case with interest for some time after, but could discern no unfavorable result from the apparently heroic practice; the bone was kept admirably in position, and every thing seemed progressing favorably. The instrument of Mon. Malgaigne, for retaining in apposition the fragments of a fractured patella, has been long before the profession. It was natural to have supposed, *á priori*, that serious inflammatory action of the fibrous tissues covering the bone, and of the bone itself, might result from its use; yet experience has not only failed to confirm such an opinion, but has assured us that, in many cases, this instrument may be very successfully employed. It may be the same, with the means above described, for the management of fractured tibia. I apprehend that the cases are few in which such practice need be brought into requisition, still the method may be a good one to have in reserve. Pertinent to this question, and as an indication of sentiment in regard to this energetic surgery, I would mention having seen at Chariere's, a day or two since, a newly invented instrument for retaining in apposition the fragments of a fractured clavicle, the principle being precisely the ame as that of Mon. Malgaigne's, for fractured patella.—*Charleston Medical Journal.*

